# Research on the financial early warning models based on ensemble learning algorithms: Introducing MD&A and stock forum comments textual indicators

**DOI:** 10.1371/journal.pone.0323737

**Published:** 2025-05-22

**Authors:** Zhiheng Zhang, Zhenji Zhu, Yongjun Hua

**Affiliations:** 1 School of Accounting, Chongqing University of Technology, Chongqing, China; 2 School of Economics and Business Administration, Chongqing University, Chongqing, China; University of Hamburg: Universitat Hamburg, GERMANY

## Abstract

This study analyzes 284 publicly listed companies first designated as ST or *ST between 2015 and 2023. It utilizes two types of textual indicators: Management’s Discussion and Analysis (MD&A) and stock forum comments. PCA and MLP are employed for dimensionality reduction. The study compares the recognition performance of single-class models with ensemble learning models while also examining the impact of various base learners and meta-learners on the performance of the ensemble learning model. The findings show that using the two types of textual indicators significantly enhanced the model’s accuracy in recognition. The single-class and ensemble learning models demonstrated average improvements of 1.24% and 1.75%, respectively. Notably, stock forum comments outperformed MD&A text. Additionally, the MLP proved more effective in feature processing than PCA. The D-M-BSA-FT model achieved an accuracy of 88.89%. Ensemble learning models outperform single classification models. After introducing textual features, the ensemble learning model achieved an average recognition accuracy of 85.31%, compared to 82.09% for the single classification model. Therefore, the financial warning model developed in this study provides valuable insights for enhancing the accuracy of financial warning identification.

## Introduction

Predicting financial distress is still an essential and unreasonable problem in current financial research. Financial dilemma refers to the company’s current cash flow not being enough to compensate for existing debt, causing the enterprise to temporarily or sustain financial difficulties [1]. Continuous financial problems will lead to financial failure and, eventually, bankruptcy for the enterprise. Early research suggested that a company is considered to be in financial distress if it experiences situations such as bankruptcy, defaulting on preferred stock dividends, being unable to repay maturing debts or overdrawing bank deposits [2]. Later, scholars further expanded the scope of financial distress in companies. For example, when a company undergoes large-scale asset restructuring, has negative net assets, is insolvent, delays payment of trade payables, or postpones the payment of maturing interest and principal, these are signs of the company experiencing financial distress [3]. Therefore, The definition of financial distress should consider the company’s actual condition and financial performance, including financial indicators and the impact of non-financial factors, to identify potential risks comprehensively.

According to the statistics of the CSMAR database, as of 2023, a total of 5381 listed A-share companies on the Shanghai and Shenzhen Stock Exchanges have been labeled as ST (special treatment). In 2021, due to consecutive losses for two years, the number of newly added ST and *ST companies in the A-share market of Shanghai and Shenzhen was 32 and 74, respectively. In 2022, the numbers were 34 and 88, respectively. In 2023, the numbers increased to 39 and 93, respectively. Generally speaking, the number of ST companies has increased significantly every year. The market risks of the listed company’s operating performance and potential market risks are worthy of attention. Suppose the early warning of a company’s financial distress can be improved from a non-financial perspective based on financial information. In that case, it may reduce the risk of investor losses due to the company’s financial difficulties to some extent. In light of this, this paper aims to construct a financial warning model that seeks to identify companies at risk of financial distress, particularly those that may be labeled as ST companies, through multidimensional analysis based on financial information. By improving the early warning mechanism for financial distress, this model can help reduce the risk of investor losses due to corporate financial crises and provide strong data support for relevant decision-making.

Research on financial early warning typically begins with identifying indicators that signal whether a company is at risk of an economic crisis in the future [4]. An increasing number of scholars are focusing on the role of text indicators in selecting warning indicators [5,6]. Researchers commonly use financial, non-financial, and individual text indicators in their studies [4]. Although some attention has been given to text-based indicators, their importance has not yet been fully recognized. Text indicators are an essential form of information disclosure, including rich language characteristics, and different types of texts provide different supplementary information. Therefore, multiple text indicators can be used as incremental information, to a certain extent, enrich the financial warning index system and help improve the accuracy of the financial early warning predictive model.

With the continuous optimization of the indicator factors, the challenges in research are becoming more complicated, especially problems such as uneven samples, increased model dimensions, data redundancy, and unrelated characteristics, which have become more prominent. Existing machine learning methods have appeared to be limited when dealing with these challenges. Related literature shows that deep learning methods can effectively solve problems such as large model dimensions, data redundancy, and characteristic irrelevant features [7].

Machine learning algorithms can effectively identify financial warnings, but their research progress has reached a bottleneck [8–11]. Some scholars have found that ensemble and deep learning algorithms perform better in financial warning identification by comparing different algorithms [12]. However, the financial early warning model based on ensemble learning and deep learning algorithms has not been established. This study has established 16 financial warning identification models based on deep learning and ensemble learning. When applying ensemble learning algorithms, most scholars have not considered the potential disagreements with pre-selected base models and meta-models and instead directly train the fixed base and meta-models. This study expands on this by comparing combinations of base models and meta-models and evaluating their performance based on Accuracy, AUC, Type I error rate, and Type II error rate. Through this comparison, we identify the optimal ensemble learning model, thereby improving the accuracy and adaptability of the financial crisis early warning model.

This study proposes a framework for efficient financial warning classification by combining dimensionality reduction, text analysis, and machine learning techniques. To increase the information dimension of text features, 2 different text feature fusion methods are employed. To reduce the high-dimensional feature space, 2 different dimensionality reduction methods were tested. This study develops 16 financial warning identification models based on dimensionality reduction, text analysis, and machine learning techniques. Additional experiments were conducted utilizing different text information to identify the most suitable text features for the ensemble learning model. All experiments performed in this study were based on data from 568 A-share listed companies in China, covering the period from 2015 to 2023. Accuracy, AUC, Type I error rate, and Type II error rate were used to evaluate the efficiency of the combinations of dimensional reduction, text features, and machine learning models.

The article is structured as follows. ‘Introduction’ presents the scope of the study, its motivation, and its contributions. ‘Literature Review’ presents a literature review on indicator selection, model construction, and dimensionality reduction in bankruptcy or financial distress prediction. ‘Algorithm description and the proposed model’ introduces the proposed theoretical framework. ‘Data’ describes the data of A-share listed companies in China used in this study. ‘Results Analysis’ summarizes the obtained results. The final conclusions are discussed in ‘Conclusions’.

## Literature review

### Selecting indicators

The choice of financial variables has always been a core issue in studying financial early warning models. Early research often relied on traditional financial ratios, such as current ratio, quick ratio, debt-to-asset ratio, profit margin, etc., to indicate a company’s financial health [12]. However, as research deepens, scholars have found that a single financial indicator is not enough to fully reflect the financial health of enterprises, so more financial variables are introduced into early warning models. For example, Leverage ratio, Return on Assets (ROA), Return on Equity (ROE), etc., have been shown to have a strong correlation with corporate default risk [13]. In recent years, with the rise of big data and machine learning technologies, researchers have begun to explore how to optimize the selection of financial variables in a data-driven way. Regularization methods such as LASSO regression are widely used in selecting financial variables, reducing redundant variables and improving the model’s prediction accuracy by restricting and punishing the model [14]. In addition, principal component analysis has also been used to reduce dimensionality and extract the most explanatory factors from numerous financial variables [15]. These methods have been widely used in international research and can effectively identify the key indicators most relevant to financial risk. In addition to traditional financial indicators, the application of text data in financial early warning models should gradually attract attention. With the development of information technology, text data such as financial reports, news reports, social media, and announcements are considered essential sources for predicting corporate risks. In particular, Schumaker and Chen argue that news texts and social media feeds in financial markets can reflect changes in market sentiment, which play an essential role in predicting a company’s financial health [16]. With the development of sentiment analysis and natural language processing (NLP) technology, researchers can extract valuable information from large-scale text data to assist in identifying financial risks [17]. For example, analyzing text data such as MD&A [18] and stock bar reviews [19,20] can capture potential risks or market reactions to an event not fully expressed in financial reports. It has been proved that the accuracy of early warning models is significantly improved by adding text information [21]. Huang effectively enhanced the model’s prediction accuracy by combining the sentiment fluctuation information in MD&A text data with traditional financial ratios [22]. In addition, text mining methods based on deep learning have also been introduced into text data analysis, which can further improve the accuracy of sentiment analysis and the prediction ability of models.

While significant progress has been made in these studies, there is still a lack of direct comparison of the effects of combining financial data with textual data compared to the results of studies using only traditional financial indicator models. For example, conventional financial ratio analysis usually predicts financial distress through current ratios, quick ratios, etc. Although these models have specific predictive capabilities, they typically have missing or limited variable information and cannot fully reflect the company’s financial risks. When text data such as MD&A or stock bar reviews are introduced, sentiment analysis and mood swings are often added to provide more sensitive signals. For example, Sun et al. significantly improved the accuracy of financial alerts when they included text data for social media comments [23].

Therefore, the selection of variables in the financial early warning model is no longer limited to traditional financial indicators, and more and more text data, especially MD&A and stock bar reviews, have begun to be incorporated into the analysis framework, and valuable information is extracted through techniques such as sentiment analysis. By combining financial variables with multiple text data, researchers can evaluate the financial health of enterprises from various dimensions and improve the accuracy and reliability of financial warnings.

### Constructing the model

The earliest Western scholars studying financial crisis warning models used the single-variable sorting method to analyze financial ratios [2]. However, the prediction accuracy was unstable due to the limited number of indicators and the poor selection of variables. To address the shortcomings of single-variable models, some scholars proposed the Z-SCORE model [12] and the O-SCORE model [13]. However, the models mentioned above still have some shortcomings, such as not considering non-financial factors, being unable to adjust their weights or factors automatically, and weak adaptability to modern corporate financial warnings. As a result, many scholars have shifted their focus to ensemble learning methods to overcome these limitations of traditional models.

With the rapid development of artificial intelligence, many scholars have increasingly focused on machine learning research. Single classifier algorithms include LR [24,25], DT [26], SVM [27], BPNN [28], and Bayesian algorithm [29].

Ensemble learning has been widely applied in the community of scholars through the continuous development of computer technology. These algorithms enhance prediction accuracy by combining multiple weak classifiers into a stronger one, thereby improving generalization ability. The most mainstream ensemble learning algorithms [30,31] include three key types: Bagging, Boosting, and Stacking.

The Bagging algorithm, first proposed by Leo Breiman, focuses on reducing overall generalization error by combining multiple models. However, Breiman found that when faced with large training sets, the algorithm’s ensemble effect can weaken [32]. Zhou Zhihua suggested integrating clustering algorithms with Bagging to improve its performance [33]. Subsequently, Zhang Chunxia summarized strategies for selecting ensemble learning algorithms [34]. However, Bagging also has limitations. Shi Jianzhong noted that Bagging can lead to overfitting, especially when dealing with noisy data [35]. A typical representative of the Bagging algorithm is RF, which integrates multiple decision trees. However, due to its susceptibility to overfitting, RF tends to be less robust, and its performance improvement is often limited. As a result, combining Bagging with other algorithms could enhance model stability.

The Boosting algorithm, like Bagging, relies on iterative training. The algorithm adjusts the data distribution in each iteration, placing more weight on misclassified samples from the previous round, thereby gradually improving model accuracy. Some popular Boosting variants include the Gradient Boosting Algorithm [36], LGBM [37], and XGBoost [38]. Zhang Lu constructed a Bagging-vote multi-source information fusion model that enhances financial early warning capabilities for listed companies [39].

Stacking, the third ensemble method, requires selecting a meta-learning model. Feeding several base learners’ prediction results into the meta-learner, an integrated model with more substantial predictive accuracy and generalization ability is obtained. By comparing the Voting and Stacking methods, the latter outperforms the former [40]. The Stacking ensemble algorithm has been applied to various domains, such as identifying malicious web pages [41], early warning of P2P online loan default risks [42], and predicting credit breaches [43]. Additionally, some scholars [44] have developed a systematic financial fraud detection model based on multi-data mining and a meta-learning framework, which has significantly enhanced the accuracy of financial fraud detection. These studies highlight the potential applicability of Stacking ensemble learning methods in the financial field and offer new directions and insights for financial crisis warning research.

### Dimensionality reduction

Technological progress and data availability will affect the development of intelligent systems. However, the increase in the number of features will have a negative impact on the model. Therefore, the application of data mining and machine learning methods in the financial sector is mainly to determine the relationship between corporate financial and financial indicators and prediction capabilities. Aghakhani et al. showed that the dimension reduction strategy played an important role when dealing with many characteristics of uncertainty relationships [45]. The maintenance method is divided into feature selection and extraction technology.

Feature selection is a dimension reduction method that removes redundant, noisy, and non-information features from the original characteristics. This method can understand its characteristics and is widely used in early financial warning research. The feature extraction method is the process of converting high-dimensional data to low-dimensional feature space. For example, the PCA and TSNE methods are used for data reduction processing in the background of financial difficulties. At present, Ye Zhengjuan has introduced a feature extraction method based on random forests, which offers high precision with minimal misclassification and fewer iterations [46]. In recent years, deep learning has occupied various fields in the community of scholars through the continuous development of computer technology. The advantage of deep learning lies in its ability to gradually learn from multiple networks to directly extract features from raw data, which can help models increase prediction accuracy. Some of the more established deep learning algorithms include MLP [47], self-organizing maps [48], BPNN [49], CNN [50], and ANN [51]. While these algorithms are more commonly used in biological sciences, scholars have also applied them in the financial field. For example, Ravisankar compared probability neural networks, multi-layer feedforward neural networks, and other machine learning models using a dataset of Chinese-listed companies [24]. The results showed that deep learning outperformed other methods regarding feature selection and predictive accuracy, highlighting its potential in financial data analysis. Similarly, BP neural networks were introduced in constructing Benford and Myer quality factors, with the new model demonstrating better prediction accuracy and stability [32]. Additionally, Cao et al. utilized the BI-LSTM model for extracting emotional characteristics from shareholder text and integrating it into the RCC parallel network, successfully achieving comprehensive learning of complex features and improving the depth and accuracy of emotional analysis [52]. These studies demonstrate that a key strength of deep learning lies in its stable and accurate output, particularly feature selection, making it a promising tool for financial analysis.

## Algorithm description and the proposed model

To better understand the basic principles of each algorithm and complete the model construction in this article, the basic principles of the BPNN, SVM, RF, AdaBoost algorithm, and Stacking ensemble algorithm adopted in this study are briefly explained below, followed by the construction of the financial warning model in this article.

### Algorithm description

#### BPNN.

**Fig 1:**
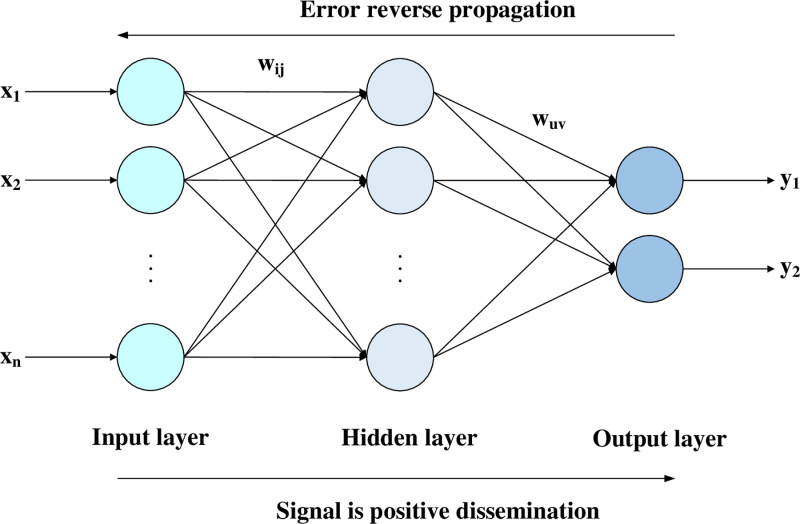
Three-layer reverse propagation neural network.

Reverse propagation neural networks are one of the foundations of deep learning models. It includes two processes: positive transmission of signals and dissemination of errors. Because of its substantial flexibility, adaptive ability, and wide application, it has been widely used in the financial sector in recent years. As shown in Fig 1 below, this is a classic three-layer BP neural network schematic diagram, including input, hidden, and output layers. The x in the figure shows the characteristics of the input, y represents the label of the output, and the hidden layer in the middle is the model training process. Among them, the weight of $\omega_{ij}$ is the weight of the input layer to the hidden layer, and $\omega_{ik}$ is the weight of the hidden layer to the output layer.

The implementation process of reverse propagation of neural network algorithms is as follows:

① Forward propagation is the transmission process from the input layer to the output layer. The core formula is as follows:

$$A_{j}=f\left(\sum_{i=1}^{n}\omega_{ij}x_{i}+b_{j}\;\right),j=1,2,\cdots ,l$$ (1)

In the formula (1), f is the activation function, $\omega_{ij}$is weight, $x_{i}$ is the output of the previous layer, and $b_{j}$ is the bias item.

② Error calculation: After obtaining the network’s output through forward propagation, it is necessary to calculate the output error, which is the difference between the network’s predicted value $O_{k}$ and the actual target value $Y_{k}$, denoted as $E_{k}$.

$$E_{k}={Y_{k}-O}_{k},k=1,2,\cdots ,m$$ (2)

③ Reverse communication: Update weight and bias.

$$\omega_{ij}=\omega_{ij}+\eta A_{j}\left(1-A_{j}\right)x_{i}\sum_{k=1}^{m}\omega_{uv}E_{k}\;,i=1,2,\cdots ,n;j=1,2,\cdots ,l$$ (3)

$$\omega_{uv}=\omega_{uv}+\eta A_{j}E_{k},j=1,2,\cdots ,l;k=1,2,\cdots ,m$$ (4)

$$a_{j}=a\left(\sum_{i=1}^{n}\omega_{ij}x_{i}+b_{j}\;\right),j=1,2,\cdots ,l$$ (5)

$$b_{k}=b_{k}+E_{k},k=1,2,\cdots ,m$$ (6)

The upper formula of ηis the learning rate, the $\omega_{ij}$ and $\omega_{uv}$ in the formula (3) and the formula (4) represent the weight update between the input layer and the hidden layer and the hidden layer and the input layer. The formula (5) and (6) indicates the offset $\alpha_{j}$ and $b_{k}$ of the hidden layer and output layer.

④ iteration training: Through multiple positive transmission and reverse propagation, BPNN has continuously adjusted the weight to gradually reduce the output error, and finally optimize the weight configuration to improve the accuracy of the model.

#### SVM.

**Fig 2:**
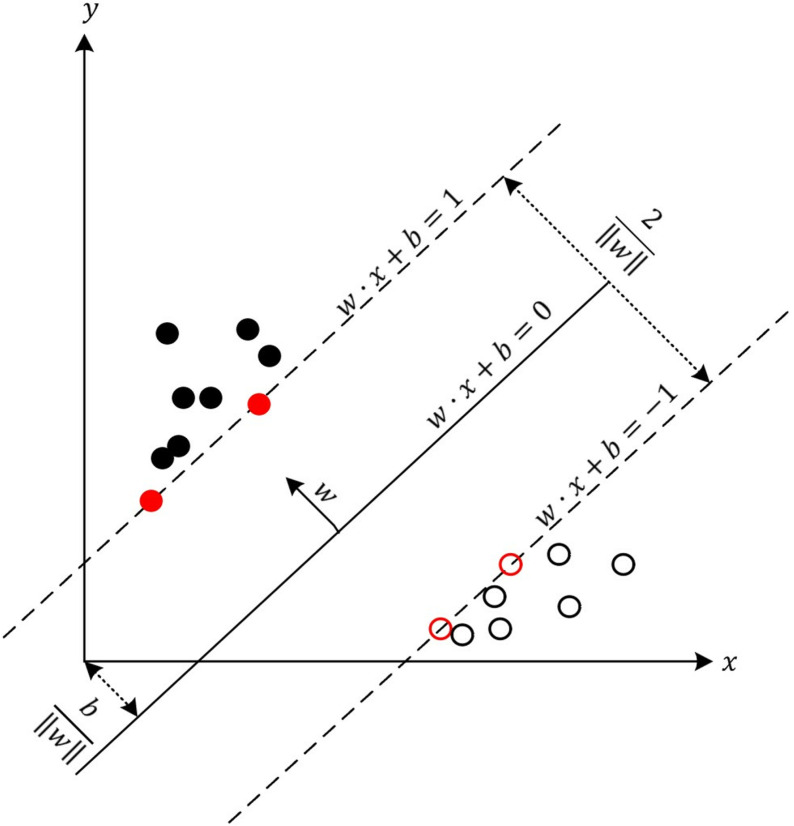
Optimal classification super plane.

SVM is a supervised learning algorithm. The basic idea is to achieve classification by finding the best ultra-flat plane (Fig 2) so that different types of samples are separated, and the edge of the two types of samples to the distance from the super-flat surface is equal and maximum. The advantage of this algorithm is that it can solve the problem of high latitudes, low samples, and non-linearly separable. In addition, when the sample data is more complicated, the nuclear function is needed. It can map the sample space to a higher dimension so that the sample can be divided linearly in the high-dimensional space. Depending on the mapping method, the types of nuclear functions are different, mainly including linear nuclear functions, polynomial nuclear functions, and Gauss nucleus functions. When predicting financial dilemma, Jones found that the SVM based on the Gaussian nuclear function was significantly better than the support vector machine with other nuclear functions [53]. Therefore, this article studies the financial crisis warning of listed companies based on the SVM of the Gaussian nuclear function.

#### RF.

The random forest algorithm is an integrated learning method. By constructing multiple decision-making trees and integrating their results, the accuracy and robustness of the model are improved. Its basic idea is to generate various training sets through the sampling method of putting back. Each training set is trained to train a decision tree. When selecting the best splitting feature, each tree randomly chooses a subset of features to reduce the correlation between trees. The final predicted class is determined through a voting process.

**Fig 3:**
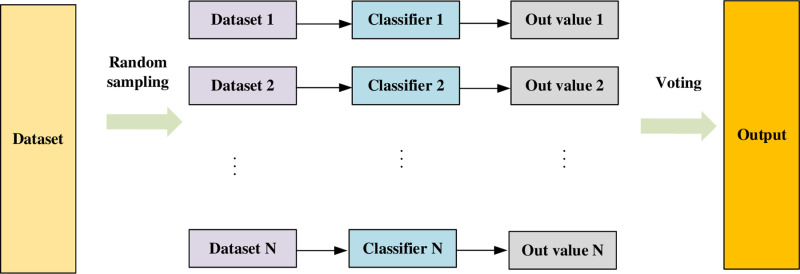
RF Algorithm Principles.

The random forest schematic Fig 3 is shown below: First, the original training set is N times a random sampling of the placement back to get N training subsets. In each sample, the probability of a single sample is $\frac{1}{n}$, and the probability of not being selected is $1-\frac{1}{n}$. After N time sampling, the likelihood of not being chosen for a sample is ${\left(1-\frac{1}{n}\right)}^{n}$. When N approaches the infinite large, this probability is close to $\frac{1}{e}$, about 36.8%. These unplugs samples are called Out-Of-Bag, which can be used to evaluate the generalization of models. Next, use this N collection to train N decision tree models respectively, then integrate the prediction results of each model through the voting method, and finally output the classification results.

#### AdaBoost.

**Fig 4:**
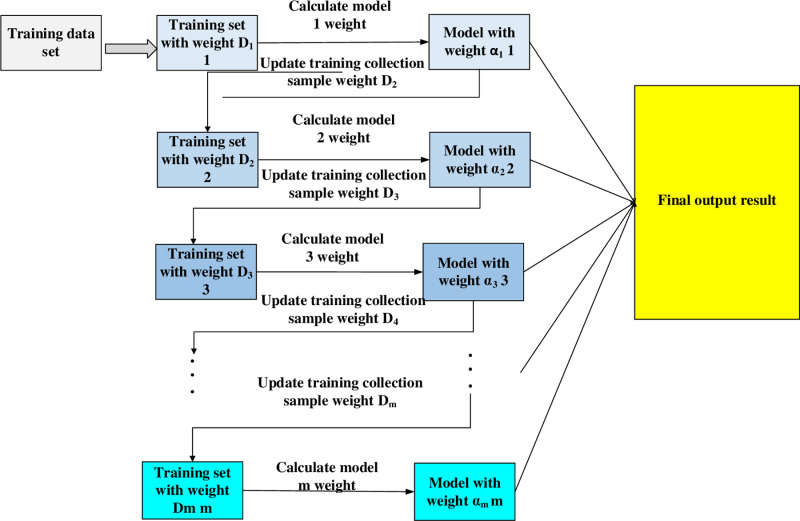
Adaboost algorithm schematic diagram.

The principle of the AdaBoost algorithm is shown below in Fig 4. The core idea is to build a more accurate overall classifier by combining multiple weak classifiers. The key is to adjust the sample weights so that subsequent weak classifiers focus more on the errors made by previous classifiers, thereby gradually improving the overall model’s accuracy until it reaches a satisfactory level.

The specific calculation steps are shown below:


$$e_{n}=\sum_{i=1}^{n}\omega_{i}\cdot I\left(h\left(x_{i}\right)\neq y_{i}\right)\;$$ (7)

$h\left(x_{i}\right)$ in formula (7) is the prediction of the sample $x_{i}$ for the weak classifier. $y_{i}$ is the actual label of the sample. I is the indicator function. It means that if the classification error is 1, it is 0.


$$\alpha_{m}=\frac{1}{2}\ln(\frac{1-e_{m}}{e_{m}}\backslash )$$ (8)

According to the error rate of the formula (7), the weight of the weak classifier in the formula (8) $\alpha_{m}$, when the weak classifier error rate is low, the $\alpha_{m}$ is significant, indicating that the classifier contributes to the final model. The weight of weak classifiers with significant errors is small.


$$\omega_{i}\leftarrow \omega_{i}\cdot \exp\left(-\alpha_{m}\cdot y_{i}\cdot h_{m}\left(x_{i}\right)\right)$$ (9)

Among them, $\exp\left(-\alpha_{m}\cdot y_{i}\cdot h_{m}\left(x_{i}\right)\right)$ represents the update of sample weights based on the classification results. The weights of misclassified samples increase, while the weights of correctly classified samples decrease accordingly. Therefore, the model can focus more on the samples that were misclassified.


$$h_{x}=sign(\sum_{i=1}^{M}\alpha_{m}h_{m}(x)\;\backslash )$$ (10)

$h_{x}$ in the formula (10) represents the final prediction category. $\alpha_{m}$ represents the weight of the weak classifier, and $h_{m}(x)$ is the prediction result of the m weak classifier.

### The proposed model

**Fig 5:**
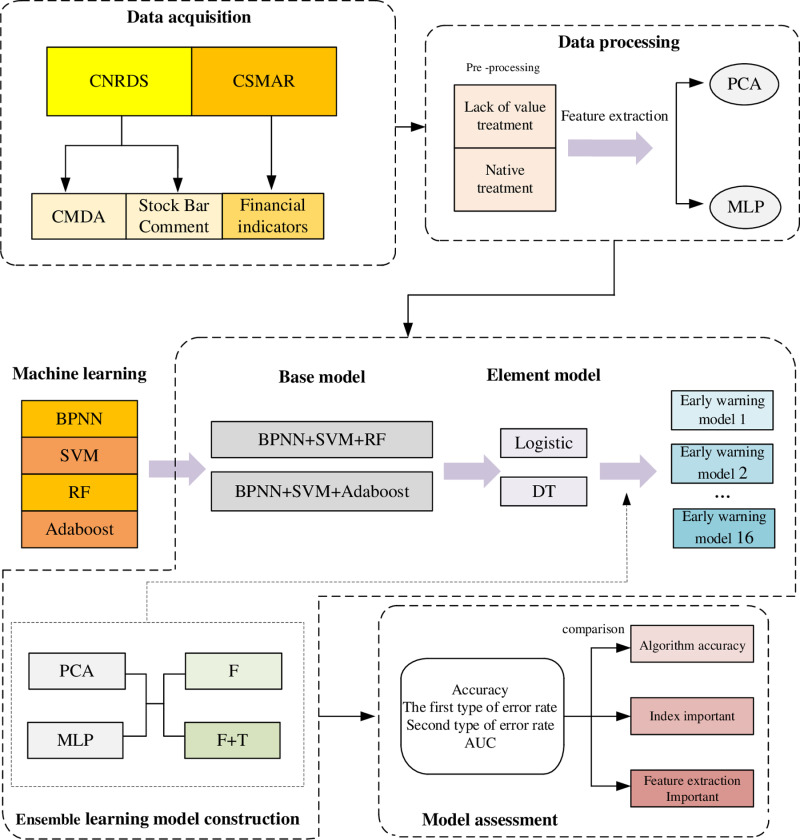
Model flow chart.

The financial early warning model designed in this article is based on deep learning feature extraction and ensemble learning framework construction. The MD&A text index and stock bar comments are introduced based on this. The implementation route is shown in Fig 5.

The base models adopted in this study include BPNN, SVM, RF, and AdaBoost. The base models’ data sources include annual report data, MD&A, and stock forum comments. The output of the base models serves as the input for the meta-model. The meta-model consists of Logistic Regression (Logistic) and Decision Tree (DT). Subsequently, the meta-model undergoes repeated training and stacking, resulting in a higher accuracy model. Therefore, the entire process, from the underlying data input and base model training to the final stacking training, is called ensemble learning.

The following part introduces the prediction process of the stacking integrated learning algorithm. Suppose there is a task T, the training data of the task is $D=\{(x_{i},y_{i}{\}}_{i=1}^{N}$, where $x_{i}$ is the input feature and $y_{i}$ is an actual label.

The training and predictions of the base learning device are expressed explicitly as:

① Assume that the K based learning device is set $f_{1}{,f}_{2},\ldots {,f}_{k}$, each base learning device is trained and predicted according to the training data. For the training set D, the prediction of each base learning device can be expressed as: ${\hat{y}}_{i}^{\left(k\right)}=f_{k}\left(x_{i}\right),k=1,2,\ldots ,K$, Among them, ${\hat{y}}_{i}^{\left(k\right)}$ is the prediction result of the base learning device $f_{k}$ on the sample $x_{i}$. Next, the prediction results of all base learning device are characterized by the characteristics of the new training set. For each sample $x_{i}$, its new feature is the prediction result of the base learning device:

$$X^{\prime }=[\begin{array}{ccc} \begin{array}{c} f_{1}\begin{array}{cc} \left(x_{1}\right) & f_{2}\left(x_{1}\right) \end{array}\\ \begin{array}{cc} f_{1}\left(x_{2}\right) & f_{2}\left(x_{2}\right) \end{array} \end{array} & \begin{array}{c} \cdots \\ \cdots \end{array} & \begin{array}{c} f_{K}\left(x_{1}\right)\\ f_{K}\left(x_{2}\right) \end{array}\\ \begin{array}{cc} \vdots & \vdots \end{array} & \ddots & \vdots \\ \begin{array}{cc} f_{1}\left(x_{N}\right) & f_{2}\left(x_{N}\right) \end{array} & \cdots & f_{K}\left(x_{N}\right) \end{array}]$$ (11)

In this way, a new feature matrix $X^{\prime }$ is the prediction result of the base learning device for each training sample.

② Training a meta-learning device by using the output of the base learning device is usually a linear regression model and logical regression model. Use a new training set $X^{\prime }$ to train the meta-learning device. The target is through the minimum target function, such as minimizing the prediction errors to get the optimal model parameter $\theta_{meta}$. For the new sample $x_{new}$, the prediction result ${\hat{y}}_{i}^{meta}$= $g\left(X_{i}^{\prime };\theta_{meta}\right)$ is obtained through the base learner, resulting in the final prediction:

$${\hat{y}}_{new}^{meta}=g\left(\left[f_{1}\left(x_{new}\right),f_{1}\left(x_{new}\right)\ldots f_{1}\left(x_{new}\right)\right];\theta_{meta}\right)$$ (12)

Under the integrated learning framework, the base model is best for choosing a weak learning device with different characteristics. BPNN is good at dealing with complex traits and non-linear issues but usually requires a lot of data to train, and the sensitivity of parameter learning rates and layers is high. SVM can make up for the disadvantages of BPNN on small data sets. It differs from BPNN because it separates the data points by looking for the best super flat plane. RF and AdaBoost are integrated learning. RF belongs to the Bagging integrated algorithm. It enhances the overall robustness by integrating multiple trees. Further, balance the lack of local optimal and parameter sensitivity. Therefore, by combining the powerful non-linear modeling capabilities of BPNN with SVM’s boundary optimization characteristics and RF’s diversity, the integrated learning model is more adaptable and robust when facing different types of data. Additionally, the AdaBoost algorithm is an ensemble boosting method. The principle is to combine multiple weak classifiers to create a strong classifier. The AdaBoost enhancement mechanism compensates for the limitations of BPNN and SVM in classifying specific complex samples, maximizing the contribution of each base model.

### Performance validation

This paper analyzes the results of the financial crisis early warning from two aspects: the single-classification model and the ensemble learning model. In terms of the single-classification model, a comparative analysis was carried out from two perspectives: dimension reduction and text index. In terms of the ensemble learning model, the selection of base learner and meta-learner is introduced based on the single classifier model, and the recognition ability of the model is comprehensively evaluated from these three perspectives. The above two aspects of research aim to explore whether MLP feature selection methods, text indicators, and ensemble learning models can effectively improve the recognition accuracy of the model.

Building a model is aimed at solving specific problems, and it typically requires some classification task metrics to evaluate the model’s performance and generalization ability. Standard model evaluation metrics include accuracy, Type I error rate, Type II error rate, and AUC value. These evaluation metrics are all based on the confusion matrix in Table 1. Based on these four evaluation metrics, this article will evaluate the model’s recognition ability.

**Table 1:** Confusion matrix.

True value	Predicted value	
The forecast is positive (1)	The prediction is a counterexample (0)
Actual positive example (1)	*TP* (TruePositive)	*FN* (FalseNegative)
Practical counterexamples (0)	*FP* (FalsePositive)	*TN* (TrueNegative)

Different evaluation indexes of the model can be obtained through the confusion matrix, and the specific evaluation indicators are explained in Table 2.

**Table 2:** Model evaluation indexes.

The name of the metric	Calculation formula	Indicator explanation
Accuracy	$\frac{TP+TN}{FP+FN+TP+TN}$	Represents the proportion of samples correctly predicted by the model to the total number of samples, which is used to measure the model’s overall performance.
AUC	$\int_{0}^{1}TPR(FPR)d(FPR)$	Refers to the area under the ROC curve, which shows the relationship between the false positive rate (FPR) and the true rate (TPR). The closer the value is to 1, the better the model output.
Type I error rate	$\frac{FP}{FP+TN}$	Among all the samples with negative values, the proportion of samples that are predicted to be positive represents the misjudgment of the model on the results of the negative examples.
Type II error rate	$\frac{FN}{FN+TP}$	The proportion of samples that are predicted to be negative examples among all samples with positive values represents the misjudgment of positive samples by the model.

### Data

#### Indicator selection.

Since too many input indicators can lead to overfitting in the model, this article builds on the research of Liu and others [54]. Based on scientific rigor, systematization, and comprehensiveness principles, 51 input variables were preliminarily selected from financial and non-financial indices. Due to space considerations, a complete list of these variables and their detailed calculation formulas is provided in the S1 Table.

#### Sample selection.

To avoid the lagged effect of financial crises and prevent overestimating the model’s predictive power, this study selects data on ST firms from 2012 to 2020, which is used to predict whether firms will experience financial crises from 2015 to 2023. To ensure rigorous comparisons, non-ST firms are selected using a strict 1:1 matching method based on the following three principles:

(1) The matched non-ST samples belong to the same industry as the ST sample, based on the 2012 industry classification standards issued by the China Securities Regulatory Commission (CSRC).
(2) The matched non-ST samples correspond to the same year of violation as the ST samples.
(3) The matched non-ST samples have an asset size similar to the ST enterprises in the corresponding year, with a deviation of no more than 30% above or below.

Following this process, 568 firms (284 ST firms and 284 non-ST firms) were identified as the study sample, as shown in Table 3.

**Table 3:** Industry Distribution of ST and Non-ST Companies.

Industry Category	ST	Non-ST	Total
Agriculture, Livestock, and Fisheries	4	4	8
Mining Industry	5	5	10
Manufacturing Industry	193	193	386
Energy Production and Supply Industry	6	6	12
Civil Engineering and Construction Industry	6	6	12
Wholesale and Retail Industry	15	15	30
Transportation and Storage Industry	3	3	6
Restaurant Industry	1	1	2
Telecommunications, Broadcasting, and Internet Services Industry	24	24	48
Property Industry	12	12	24
Leasing and Business Services Industry	7	7	14
Ecology, Environment, and Public Facility Management Industry	3	3	6
Health	1	1	2
Media and Cultural Arts Industry	3	3	6
Comprehensive	1	1	2
Total	284	284	568

Based on the firms identified as the study sample in Table 3, this study extracted two types of textual data from the China National Research Data Service (CNRDS) database: MD&A and stock bar reviews. All the study samples contain stock bar review text. These stock bar reviews cover a full year of data for ST firms and their matched non-ST firms, ensuring consistency in the time dimension. In addition, the matching process also improves data comparability by controlling for industry type and asset size deviations within 30%. Within the constraints of these matching criteria, the stock bar reviews can be effectively applied to ST firms and their matched non-ST firms, providing a strong basis for comparability in the study.

After 1:1 matching ST and non-ST companies, we found that 14 of the 51 financial indicators initially identified had many missing values. To ensure the study’s results’ reliability and avoid the negative impact of missing data on the sample size, we decided to filter out these indicators and ultimately retain 37 valid financial indicators, as shown in Table 4.

**Table 4:** 37 financial indicators finalized.

Dimension	Indicators
Debt repayment	X1;X2;X3;X4;X5;X6
Operating ability	X7; X8; X9;X10;X11;X12;X13
Profitability	X15;X16;X17;X18;X19
Development ability	X20;X21;X22;X23
Cash flow index	X25;X26;X27;X28;
Per share	X29;X30;X31
Relative value indicator	X34
Non-financial indicator	X36;X37;X38;X39;X40;X41;X42

#### Text analytics.

**Fig 6:**
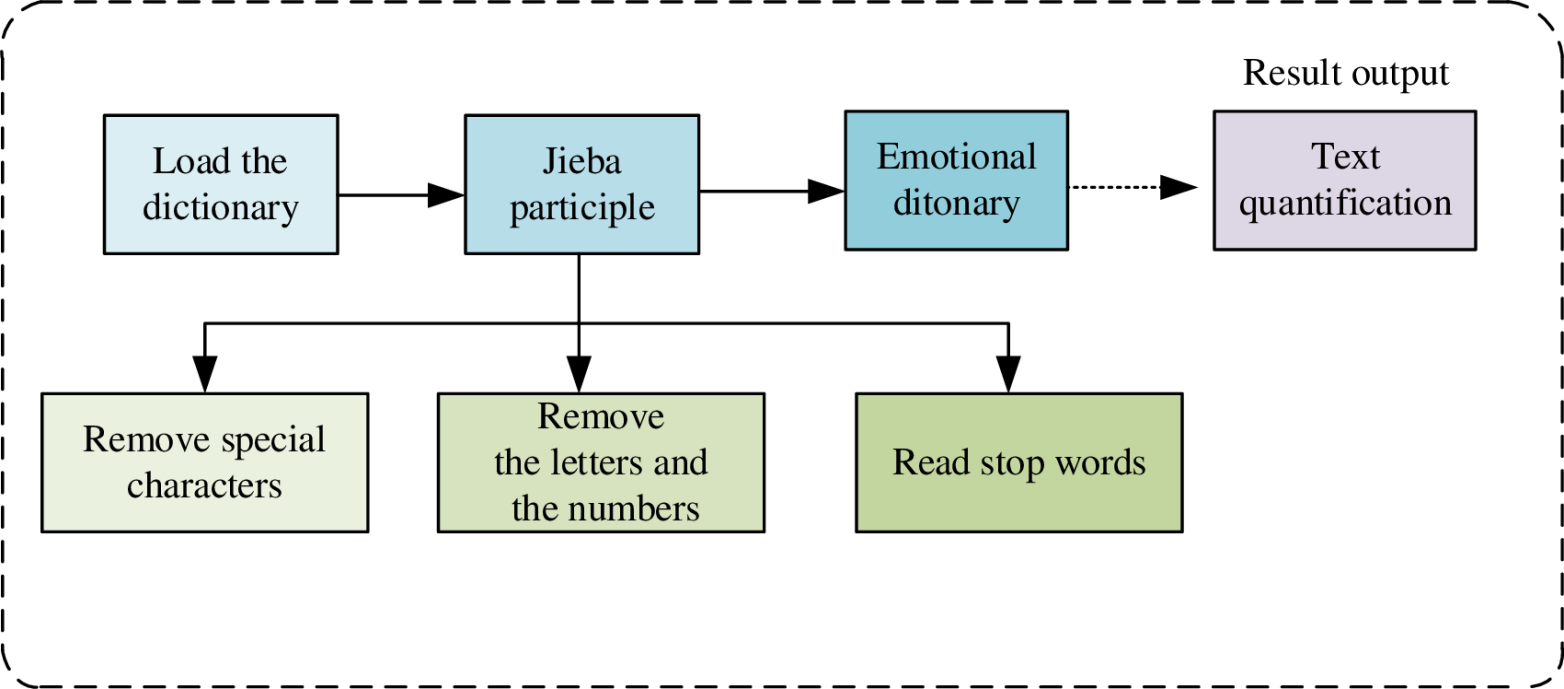
Text analysis and indicator calculation process.

In Fig 6 and Table 5, regarding text analysis, there are three main steps: load a custom dictionary, use the jieba word segmentation module in Python to segment the text, and finally output the result; the reason why we want to load the custom dictionary here is that the dictionary that comes with the jieba word segmentation module is not representative, and he will divide the text too scattered. Still, we want to divide it into one word. For example, the main business income will be divided into three words: main, business, and income, but the research hopes to divide it into one word. It is enough to add the main business income to the dictionary now. Word segmentation is mainly divided into the following three steps: (1) Read stop words because the mood particles in the text have no information value, so they are removed in the text analysis; (2) Remove numbers, letters, and special characters, which are not text content and will not be convenient for word segmentation, so remove them; (3) Perform a word segmentation operation, and then make a judgment, if the word is not in the stop word, save its output to a list. Finally, based on the bag-of-words method, such as the positive emotion dictionary, which contains words that can represent positive, by comparing the separated words with the emotion dictionary, you can know the number of positive words in this text, and then divide it by the total number of words to get a relative number, and finally quantify the text.

**Table 5:** Selection and quantification of text indicators.

The type of text	Dimension	Index	Variable definitions
Annual report MD&A text	Emotional polarity	Positive	PosWords/MD&AWords
		Negative	Neg/MD&AWords
	Text readability	Terminology	ProTermWords/MD&AWords
		Average sentence length	Length/SentenceNumbers
Stock forum comments text	Emotional inclinations	Positive emotions	PosWords/Stock CommentaryWords
		Neutral emotion	NeuWords/ Stock CommentaryWords
		Negative emotions	NegWords/ Stock CommentaryWords
	Transmissibility	Readings	Reading popularity

### Pre-processing

After obtaining the original data, this article has been processed as follows

#### Remove the missing value sample.

When the sample is pre-processed, a small number of listed companies labeled as ST or *ST due to abnormal financial conditions are first identified. ST companies with too many missing data are screened out. Since there are few such ST companies, they can be screened directly. Through analysis, it was found that the distribution of missing values was relatively uniform. If one company has many missing values, other companies with similar values may have similar conditions. This study removed these samples with too many missing values to ensure the analysis results’ representativeness and statistical significance. Due to the small number of samples deleted, the data that was eventually retained met the completeness and accuracy requirements required for analysis. Finally, according to the industry, asset size, and the matching principle of the same year, the 1:1 ratio was used to match the ST samples.

#### Feature encoding.

Many scholars have used label coding or unique codes in the financial crisis early warning research field due to the small distinguishing characteristics between ST companies and non-ST enterprises. Since label coding is simple, easy to implement, and produces better results, this article uses label encoding, marking ST or *ST samples as 1 and non-ST samples as 0.

#### Normalization.

Due to the extensive range of sample data, it is impossible to perform data analysis directly. First, text-based indicators need to be quantified, such as audit opinion types, whether the top ten shareholders have related-party relationships, whether the chairman and CEO hold concurrent positions and the nature of equity ownership. Next, data such as stock codes, industry types, the year the company was marked as ST, and labels, which do not require normalization, should be removed. Finally, normalization is performed on the remaining data. The data normalization method used in this article is Min-Max Normalization. The normalization process is based on $X=\frac{X-X_{\min}}{X_{\max}-X_{\min}}$. $X_{max}$ and $X_{\min}$ represent the maximum and minimum values of feature X, respectively. Then the sample data $X_{1},X_{2},X_{3}$ ···, $X_{n}$ zoom to the range of [0, 1].

The normalization operation ensures that the financial variables are within the same range and avoids the excessive influence of some variables with large values on the model. This is an essential step in ensuring equal weighting of financial indicators. In addition, the normalization method in this paper does not involve variable dimensionality reduction or the processing of variable correlation. Still, it ensures that the data range of different features is within the range of [0–1] to improve the robustness of the model.

#### Dimension reduction.

Data reduction is an important technique that converts high-dimensional data into low-dimensional data while attempting to retain the original information. This is particularly useful for handling large-scale datasets, as it helps reduce the need for computing resources and improves the efficiency of algorithms. The most commonly used data reduction techniques mainly include the following two categories:

(1) Feature selection

Feature selection is a method of dimension reduction, focusing on removing redundant, noisy, and non-information features from the concentration of original features. This method is standard in predicting financial difficulties because it can achieve a comparison of characteristics. It mainly includes the following four methods: ①Filtering method analyzes each characteristic to evaluate the importance of features. For example, the CFS algorithm can effectively predict the category by ensuring the selection characteristics and no redundant information between the features, thereby optimizing the selection process. ②The package method is based on a specific machine-learning algorithm to evaluate the performance of different feature subsets [55]. The principle mainly uses greedy search strategies to assess the effectiveness of various feature subsets and select appropriate performance indicators based on the task type. ③The embedded method combines the advantages of the filtering and packaging methods and allows these two methods to conduct feature selection and model training in parallel. The MLP used in selecting financial early warning characteristics is one of the technologies. This technology has reduced its coefficient to zero to eliminate predictive factors without information in the model. ④Mixing legal, mainly based on the above method.

(2) Feature extraction

Feature extraction is the process of converting high-dimensional data to low-dimensional feature space. This method includes two aspects, which are linear and non-linear methods. ①The linear method reduces the number of dimensions by using linear functions, such as the primary component analysis method (PCA), and its main idea is to map the N-dimensional symbol to the K-dimension (N > K). The new structure creates the K-dimension symbol, which is called the main ingredient. ② The advantage of non-linear methods is that they can better represent data in the real world because it is often non-linear, not linear.

According to the comparison of the feature selection and extraction method provided by Table 6, it can be seen that comprehensive and high-performance feature extraction cannot be separated from high investment calculation costs. MLP and PCA with lower time complexity are used as the dimension reduction method of this article, which also meets this article—the final research purpose.

**Table 6:** Character selection and extraction comparison.

Dimensionality Reduction	Basic Comparison	Time Complexity	Performance	Applicable to Big Data	Advantages	Disadvantages	Method	References
Feature Selection	Filter Methods	Low	Low	Very High	Low computational cost, short runtime, and low risk of overfitting.	Low performance ignores dependencies between features and does not interact with the classifier.	CFS, Relief-F, K-W	[56–58]
	Wrapper Methods	Medium	High	High	High performance, searches for feature dependencies, interacts with the classifier.	High computational cost, long runtime, and high risk of overfitting.	Backward Wrapper Method, Stepwise DA, GA, PDC-GA	[59–61]
	Embedded Methods	Low	Medium	High	Short runtime, low risk of overfitting.	Performance is poor when the number of features is small.	LASSO, XGBoost, MLP	[47,62,63]
	Hybrid Methods	High	High	High	High performance.	Feature selection depends on the classifier.	Hybrid Approaches	[64]
Feature Extraction	Linear Feature Extraction	Low	Medium	Low	Low computational cost, short runtime.	Poor performance on big data information loss.	PCA, NPE, RSL	[65,66]
	Non-linear Feature Extraction	High	High	High	Excellent performance on big data.	High computational cost, long runtime.	tSNE, SOM, Autoencoder	[67–69]

This study first performs the principal component analysis of the data. First, the Kolmogorov-Smirnov test (K-S) checks whether the sample obeys the normal distribution. The income of the inspection is gradually significantly less than 0.05, indicating that the indicator does not follow the normal distribution. The next step is tested by Mann-Whitney U. According to the results, 11 indicators such as X10, X13, X20, X23, X25, X27, X34, X37, X40, X41, X42 from Table 4 have no significant differences in ST and non-ST samples and should be deleted. Then, the Kaiser-Meyer-Olkin (KMO) and Bartlett ball test is performed; the purpose is to check whether the data after the removal is suitable for factor analysis. The results indicate that the KMO value is 0.73, more significant than 0.5, and the significance level is 0.000, less than 0.05, suggesting that the data is suitable for factor analysis. However, the KMO value of 8 text indicators is 0.5, which is unsuitable for factor analysis. Therefore, this study directly inputs 8 text indicators as indicators, which are derived from Table 5. It can be seen from Table 7 that the characteristic value of 11 ingredients is greater than 1, and its cumulative variance is also greater than 1. The cumulative contribution rate is 76.22%, indicating that the 11 main components have good representativeness and explanation. The results of the factor analysis are quite satisfactory. Therefore, this study replaces these 11 main components as input and expresses them as F1, F2,..., and F11.

**Table 7:** General Quan Differential Explanation.

Component	Initial eigenvalues			Extraction sums of squared loadings		
	Total	% of variance	Cumulative %	Total	% of variance	Cumulative %
1	4.53	17.423	17.423	3.968	15.263	15.263
2	3.223	12.395	29.818	2.737	10.528	25.79
3	2.243	8.627	38.445	2.357	9.065	34.855
4	1.817	6.987	45.432	2.281	8.772	43.627
5	1.495	5.75	51.182	1.592	6.121	49.749
…	…			…	…	…
10	1.031	3.83	72.505	1.044	4.015	72.218
11	1.017	3.716	76.221	1.041	4.003	76.221
12	0.852	3.275	79.497			
13	0.829	3.187	82.684			
…	…					
26	0.019	0.075	100			

At the same time, this study also uses an MLP as a feature extractor. MLP, also known as an ANN, is characterized by having multiple neurons and hidden layers in between, in addition to the input and output layers. Its strength lies in its ability to learn representations from training data. During model training, the algorithm identifies the loss cost for each node based on the actual activation. It adjusts the weights accordingly, which is also called the backpropagation algorithm. This study uses Python with libraries such as pandas and torch to complete the analysis, ultimately selecting 11 features. As shown in Table 8, after feature extraction with MLP and classifying the samples, the accuracy of crisis samples is relatively high. Therefore, using an MLP in deep learning for feature extraction demonstrates a certain level of reliability and accuracy.

**Table 8:** Classification performance metrics of MLP.

Label	Precision	Recall	F1-score	Support
0	0.78	0.80	0.79	284
1	0.80	0.78	0.79	284

### Model training

This paper constructs different ensemble learning models based on the selected SVM, BPNN, RF, and AdaBoost learners based on the ensemble learning framework. During model training, you need to specify model parameters. In this paper, the gradient descent method is selected to adjust the model’s parameters and maximize the accuracy of each model. Based on previous experience, this paper selects C, gamma, hidden layer n, n_estiamtors, max_features, max_depth, min_samples_split, min_samples_leaf, and learning_rate as the parameter tuning objects, and finally obtains the parameter values in Table 9 after iterative training:

**Table 9:** Base learning device parameter value.

Model	Feature extraction	PCA		MLP	
	index	F	F + T	F	F + T
SVM	Parameter C	0.060 061 054	2.862 863 577	1.521 522 369	0.020 021 018
	Parameter gamma	0.160 161 144	0.010 011 009	1.671 672 505	0.030 031 027
BPNN	Hidden layer n	5	6	7	8
RF	n_estiamtors	167	126	14	40
	max_depth	23	46	19	159
	min_samples_split	7	18	16	10
	min_samples_leaf	2	6	5	2
AdaBoost	n_estiamtors	159	287	9	10
	learning_rate	0.003 003 004	0.029 029 03	0.139 139 14	0.112 112 113

## Results analysis

### Single classification model

To verify the effects of deep learning algorithms and text indicators to improve the early warning identification of the financial crisis, this section compares and analyzes from two aspects: dimension reduction and text indicators.

#### Dimension reduction.

**Fig 7:**
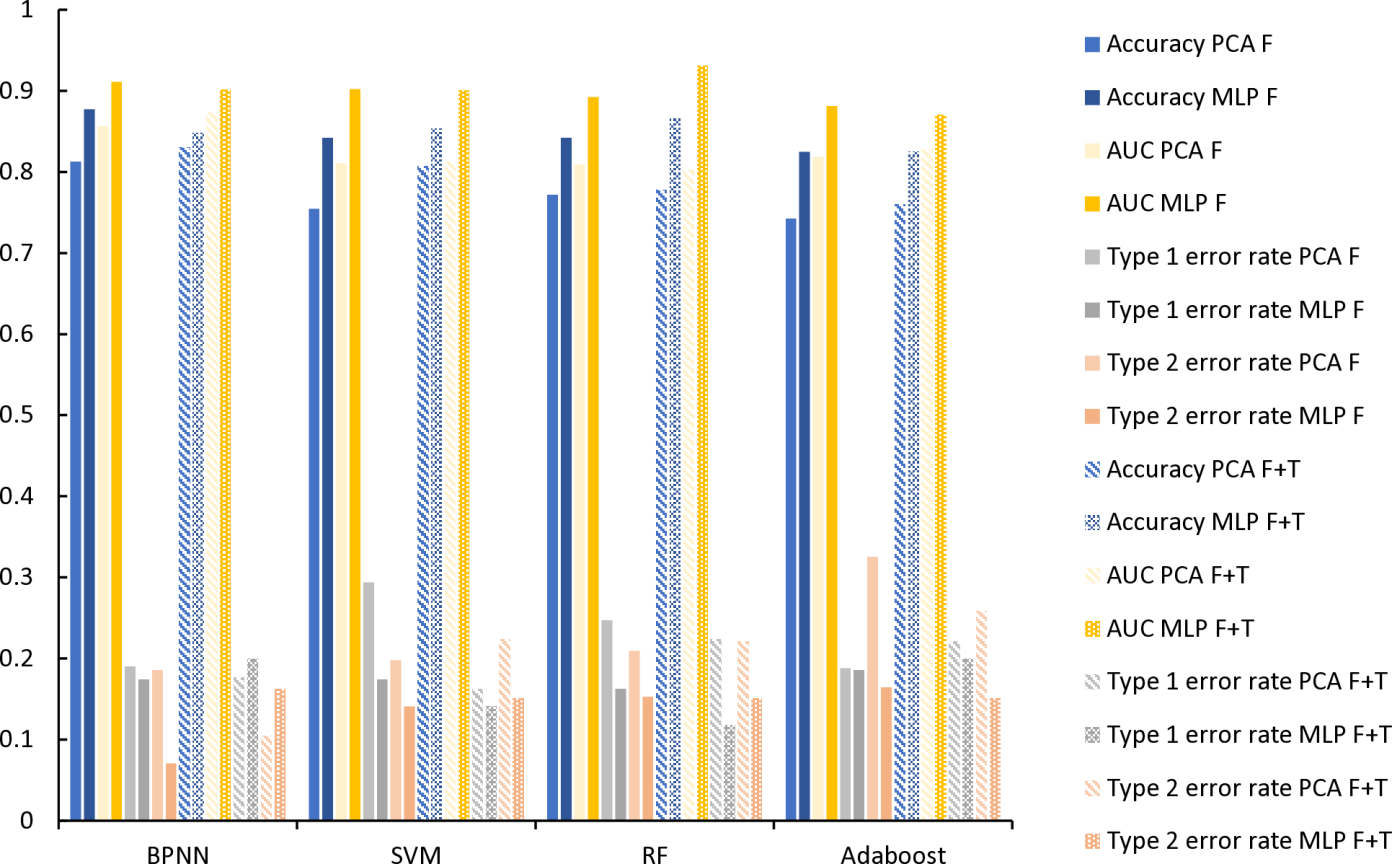
Single classification model results comparison diagram.

In Fig 7 and Table 10, the results showed that the MLP algorithm with deep learning significantly improved the accuracy of the financial crisis warning model. After using MLP for feature selection, the performance of all single classifier models has been enhanced to varying degrees, of which the AUC value of BPNN and SVM exceeds 90%. Specifically, the accuracy of BPNN reached 87.72%, an increase of 4.57% over the same model using the PCA method. In addition, the first and second types of error rates have also decreased accordingly. This significant performance improvement shows that the MLP algorithm can improve the model’s predictive accuracy under the framework of deep learning and provide more reliable support for the financial crisis warning.

**Table 10:** Different dimension reduction of a single category model prediction result.

Dimension reduction	Model	Financial			
		Accuracy	AUC	Type I error rate	Type II error rate
PCA	BPNN	81.29%	85.62%	19.05%	18.60%
	SVM	75.44%	81.07%	29.41%	19.77%
	RF	77.19%	80.94%	24.71%	20.93%
	AdaBoost	74.27%	81.85%	18.82%	32.56%
MLP	BPNN	87.72%	91.11%	17.44%	7.06%
	SVM	84.21%	90.23%	17.44%	14.12%
	RF	84.21%	89.23%	16.28%	15.29%
	AdaBoost	82.46%	88.13%	18.60%	16.47%

#### Text indicator.

In Fig 7 and Table 11, it can be seen that after incorporating the text indicators of the MD&A and stock comments, the accuracy of the four models based on PCA feature extraction has increased by 5.26%. Under MLP feature selection, the accuracy changes of the four models are a decrease of 2.93%, an increase of 1.17%, an increase of 2.34%, and no change, respectively. Although the recognition accuracy of the model under the PCA method is lower than that of the MLP method, the results of all single-classification models are improved after the introduction of text indicators. This indicates that the inclusion of text indicators positively impacts the accuracy of the financial crisis warning model.

**Table 11:** Integrated the single classification model prediction results of the text index.

Dimension reduction	Model	Financial + Text			
		Accuracy	AUC	Type I error rate	Type II error rate
PCA	BPNN	83.04%	87.32%	17.65%	16.28%
	SVM	80.70%	81.23%	16.28%	22.35%
	RF	77.78%	80.27%	22.35%	22.09%
	AdaBoost	76.02%	82.75%	22.09%	25.88%
MLP	BPNN	84.79%	90.11%	20.00%	10.47%
	SVM	85.38%	90.09%	14.12%	15.12%
	RF	86.55%	93.08%	11.76%	15.12%
	AdaBoost	82.46%	87.03%	20.00%	15.12%

According to the above analysis, MLP is more advantageous than the PCA method in terms of dimension reduction. In terms of text indicators, the results show that the introduction of text indicators can further enhance the accuracy of the early warning, especially when combining the MLP feature selection method. Combining the two types of text indicators and deep learning algorithms provides adequate incremental information for financial crisis warning recognition.

### Ensemble learning model

Traditional financial crisis recognition models make it difficult to fully use information from various feature combinations, especially when it involves mixed features involving text and financial data. In contrast, ensemble learning models have the advantage of combining multiple single-class models and can better adapt to the complexity and diversity of data. Therefore, based on comparing the characteristics of traditional models and text indicators, this article further explores the effects of different base learning and meta-learning devices on identification results.

#### Dimension reduction.

**Fig 8:**
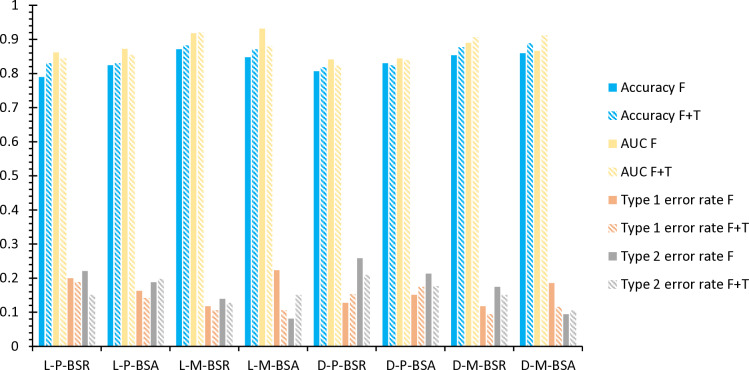
Integrated learning model results comparison diagram.

In Fig 8 and Table 12, in terms of dimension reduction methods, this article combines the two methods of PCA and MLP to obtain the following results: When the feature extraction method selects PCA, the integrated learning device is logistic, and the accuracy of the L-P-BSR-F is 78.95%, less than 80%; When the integrated learning device is DT, the accuracy of the D-P-BSR-F and D-P-BSA-F is greater than 80%, which are 80.70%and 83.04%, respectively. However, compared to using MLP as a feature selection, the results obtained from PCA are not good. When the integrated learning device is arbitrarily one of Logistic and DT, the accuracy of the model extraction of MLP as a dimension reduction is greater than 85%, up to 87.13%, and the highest is 87.13%. Although the accuracy of L-M-BSA is only 84.80%, its AUC value of 93.14% makes up for the lack of accuracy. Therefore, by comparing the performance of MLP and PCAs under different meta-model frameworks, it can further verify that the MLP feature selection method can improve the model’s overall performance. Therefore, this article selects MLP for dimension reduction.

**Table 12:** Ensemble learning model prediction results under different dimensionality reduction methods.

Meta-learner	Dimension reduction	Founder	Model	Financial			
				Accuracy	AUC	Type I error rate	Type II error rate
logistic	PCA	BPNN+SVM + RF	L-P-BSR-F	78.95%	86.16%	20.00%	22.09%
		BPNN+ SVM+ Adaboost	L-P-BSA-F	82.46%	87.24%	16.28%	18.82%
	MLP	BPNN+SVM + RF	L-M-BSR-F	87.13%	91.79%	11.76%	13.95%
		BPNN+SVM +Adaboost	L-M-BSA-F	84.80%	93.14%	22.35%	8.14%
DT	PCA	BPNN+SVM + RF	D-P-BSR-F	80.70%	84.13%	12.79%	25.88%
		BPNN+SVM +Adaboost	D-P-BSA-F	83.04%	84.45%	15.12%	21.33%
	MLP	BPNN+SVM + RF	D-M-BSR-F	85.38%	88.99%	11.76%	17.44%
		BPNN+SVM +Adaboost	D-M-BSA-F	85.96%	86.63%	18.60%	9.41%

#### Model contrast.

In Fig 8 and Table 12, the choice of different meta-learners has varying effects on the accuracy of the financial crisis warning model. When the meta-learner is logistic, the accuracy of the combined model L-M-BSR-F is 87.13%, higher than L-M-BSA-F at 84.80%. Although the accuracy of AdaBoost is lower than that of RF, its AUC value is higher, reaching 93.14%, effectively compensating for the lower accuracy. On the other hand, when the meta-learner is DT, the accuracy and AUC values of the two different combined models are 85.38% and 85.96%, 88.99% and 86.63%, respectively. Among them, the combined model D-M-BSA-F achieves the best accuracy. By comparing these results, when the meta-learner is logistic, the L-M-BSR-F combined financial crisis warning model performs the best in accuracy. In contrast, the D-M-BSA-F combined model performs second when the meta-learner is DT, with an accuracy difference of 1.17%. Therefore, Logistic as the meta-learner yields better results.

#### Text indicator.

In Fig 8 and Table 13, this study has constructed 16 financial crisis early warning models, 8 of which only use financial indicators, and the other 8 combine financial and text indicators. The core purpose of research is to verify whether the model of fusion text information can improve the accuracy of the financial crisis forecast. The model accuracy of all fusion text indicators is better than a model with only financial indicators. Among them, the accuracy of the L-P-BSR model increased from 78.95%at the beginning to 83.04%, an increase of 4.09%. The results show that text indicators can not only optimize the accuracy of identifying single classification models but also improve the prediction effect in the integrated learning model, further verifying the improvement of the text indicators on the financial crisis warning model, especially in the L-P-BSR model—the most fully reflected.

**Table 13:** Ensemble learning model prediction results based on text indicators.

Meta-learner	Dimension reduction	Founder	Model	Financial +Text			
				Accuracy	AUC	Type I error rate	Type II error rate
logistic	PCA	BPNN+SVM + RF	L-P-BSR-FT	83.04%	84.45%	18.82%	15.12%
		BPNN+SVM+Adaboost	L-P-BSA-FT	83.04%	85.43%	14.12%	19.77%
	MLP	BPNN+SVM + RF	L-M-BSR-FT	88.30%	92.05%	10.59%	12.79%
		BPNN+SVM+Adaboost	L-M-BSA-FT	87.13%	87.92%	10.59%	15.12%
DT	PCA	BPNN+SVM + RF	D-P-BSR-FT	81.87%	82.27%	15.29%	20.93%
		BPNN+SVM+Adaboost	D-P-BSA-FT	82.46%	83.97%	17.44%	17.65%
	MLP	BPNN+SVM + RF	D-M-BSR-FT	87.72%	90.63%	9.41%	15.12%
		BPNN+SVM+Adaboost	D-M-BSA-FT	88.89%	91.18%	11.63%	10.47%

#### Further analysis.

**Fig 9:**
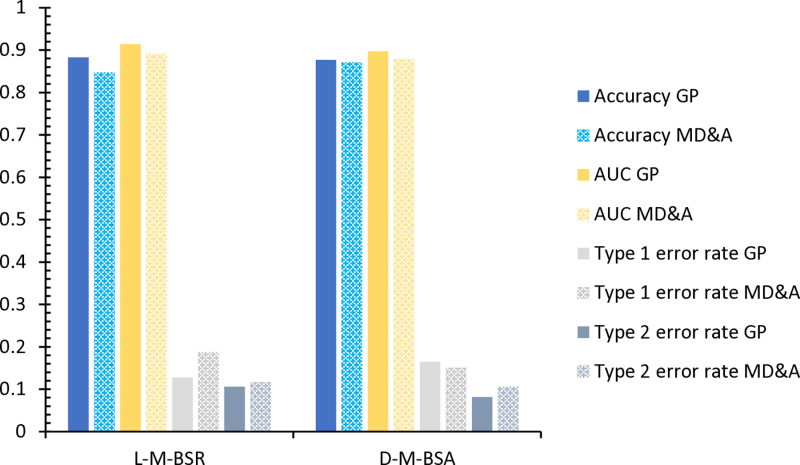
The text indicators further analyze the diagram.

Based on the above research in Fig 9 and Table 14, this article further discusses the performance of the two text indicators of the stock bar review and MD&A in the financial crisis warning model. By using the L-M-BSR-FT and D-M-BSA-FT models to compare these two text indicators, the research found that the effect of stock comments in the financial crisis forecast is better than MD&A. Specifically when the accuracy and AUC values are used as evaluation indicators the accuracy of the stock comments in the L-M-BSR-FT and D-M-BSA-FT models is 88.30% and 87.72%, respectively, which is higher than the MD&A. The accuracy rate is 84.8% and 87.13%. In addition, the performance of the AUC value of the stock bar is better than that of the MD&A. The AUC values of the two models under the stock review are 91.4%and 89.75%, respectively, significantly higher than the 89.17%and 87.94% of the MD&A.

**Table 14:** Further analysis of text indicators: prediction results of ensemble learning models.

Model & Index	Model	Accuracy	AUC	Type I error rate	Type II error rate
Logistic-MLP-BPNN+SVM + RF -Stock Forum	L-M-BSR - Stock Forum	88.30%	91.40%	12.79%	10.59%
DT-MLP-BPNN+SVM + AdaBoost -Stock Forum	D-M-BSA - Stock Forum	87.72%	89.75%	16.47%	8.14%
Logistic-MLP-BPNN+SVM + RF -MD&A	L-M-BSR -MD&A	84.80%	89.17%	18.82%	11.63%
DT-MLP-BPNN+SVM + AdaBoost - MD&A	D-M-BSA -MD&A	87.13%	87.94%	15.12%	10.59%

Therefore, the above comparative analysis shows that MLP performs better than PCA in the dimension reduction methods, regardless of whether text indicators are considered. Based on the choice of MLP for dimension reduction, the optimal combined model is L-M-BSR-FT, with an accuracy of 88.3%. The study indicates that including text indicators effectively improves the model’s recognition accuracy, with stock forum comments being particularly helpful in enhancing the model’s recognition performance.

Essentially, the above results have been further supported by verifying the four independent models, all of which indicate that the stock comments have more substantial capabilities in predicting the financial crisis and can more effectively identify potential risks. The reason is that MD&A texts usually follow fixed templates and structures, mainly focusing on financial data and operating conditions. Although such standardized reports help provide consistent information, they often lack in-depth analysis of market sentiment and investor expectations, which limits their predictive power. In contrast, the stock review text has more substantial subjectivity. Writers based on financial data and considering market dynamics, industry trends, and investor psychology. Capture potential risks and opportunities, thereby reflecting richer market emotions. Therefore, the market sentiment contained in the shares bar comment and investors expect more sensitivity to the financial crisis warning and can provide more reliable support for decision-making.

As for the predictability of stock forum commentary, this article argues that there are many reasons behind it. First, stock forum commentary typically contains much immediate feedback from investors and market participants, reflecting the market’s mood swings and collective psychology, which is often more timely and diverse than MD&A texts. Secondly, the stock forum commentary discusses the company’s financial health and views the macroeconomy, industry trends, and policy changes, which can profoundly affect the company’s future development. As a result, stock forum reviews are more forward-looking and can perceive potential risks in the market than traditional financial reports.

The potential limitations of stock forum commentary are also discussed in this article. As stock forum comments are informal data sources, they may contain subjective opinions, emotional reactions, or biases, especially when volatile market sentiment. This can lead to excessive optimism or pessimism about the business by some investors, affecting the accuracy and reliability of the reviews. In addition, the opinions in the forum comments are often not professionally analyzed or verified, increasing the information’s asymmetry. So, while stock forum reviews have certain advantages in predicting financial crises, one must be careful of their potential biases and limitations when relying on such data sources for decision-making.

Future research can further analyze the following specific factors, such as changes in public opinion, to make stock forum reviews more predictive. Perhaps by combining more quantitative analysis methods, one can effectively verify the predictability of comment content. At the same time, attention should be paid to reducing the bias in informal data sources to avoid misjudgments and decision-making biases.

### Comparison between ensemble learning and single classifier algorithms

Based on the above analysis, it can be concluded that ensemble learning models outperform single-classifier models in terms of recognition performance.

When only considering financial indicators, the average accuracy of the ensemble learning model is 83.55%, while the average accuracy of the single classifier model is only 80.85%. This indicates that ensemble methods, by combining multiple models, provide better performance, thereby improving the model’s generalization ability and predictive reliability.

When text indicators are introduced, the performance gap between the ensemble learning model and the single classifier model further widens. The average accuracy of the ensemble learning model increases to 85.31%, while the single classifier model’s average accuracy is 82.09%. This shows that ensemble models can effectively integrate different data types, enhancing their overall performance.

Despite their higher complexity, ensemble learning models can significantly improve accuracy from the perspective of performance improvement, especially when dealing with multiple data types (such as financial and text indicators). Therefore, in financial warning, the performance enhancement provided by ensemble learning models justifies their complexity.

## Conclusion

This study builds a financial warning model based on deep learning and integrated learning algorithms and is a research sample based on listed companies with ST or*ST for the first time from 2015–2023. In terms of indicators, this article introduces the text indicators of MD&A and stock bar reviews based on traditional financial variables to verify whether these two types of text indicators provide incremental information for improving financial early warning identification. Use PCA and MLP methods when dimension reduction is aimed at exploring which method yields better dimension reduction results. From this, the following conclusions are obtained: First, compared with the commonly used PCA method for feature extraction, the MLP method based on deep learning performs better in this study. The results show that, whether using financial indicators or after introducing text indicators, the MLP model consistently achieves an accuracy rate exceeding 85%, significantly outperforming the PCA method. Second, after introducing text indicators, the model accuracy rate is significantly improved compared to only financial indicators. The accuracy of single-class models increased by an average of 1.24% after adding text indicators, of which the SVM model was increased by 5.26%. The integrated learning model increased by 1.75%, of which the L-P-BSR model increased by 4.09%. In addition, through further analysis, the results show that in the L-M-BSR and D-M-BSA models, stock forum comments have a more significant impact on improving model accuracy. Furthermore, when both text indicators are used together, the model’s accuracy is higher than when only one text indicator is used. Third, the identification effect of integrated learning models is better than a single classification model. When considering only financial indicators, the average accuracy of the integrated learning model is 83.55%, while the average accuracy rate of the single classification model is only 80.85%. After introducing text indicators, the average accuracy of the ensemble learning model is 85.31%, while the average accuracy of the single classification model is 82.09%. This further proves that text indicators help improve the model’s recognition effect. This study further explores the impact of different base and ensemble learners on the model. The results show that the L-M-BSR model performs the best, with an accuracy rate of 88.3%, which is 6.43% higher than the lowest accuracy rate of similar models.

## Supporting information

S1 Table Primary financial warning identification indicators (financial and nonfinancial indicators). (DOCX)

## References

[1] Sun J, Zhang Y, Li X, Wang H, Zhou J. Class-imbalanced dynamic financial distress prediction based on adaboost svm ensemble combined with smote and time weighting. Inf Fusion. 2020;54:128–44.

[2] Beaver WH. Financial ratios as predictors of failure. J Account Res. 1966.

[3] Sun J, Li N, Zhao M. Negative media coverage and corporate financial distress warning: based on text analysis and machine learning. Financ Econ Rev. 2023;09:80–90.

[4] Md Nasir NAB. Real earnings management and financial statement fraud: evidence from Malaysia. Int J Account Inf Manag. 2018;26(4):508–26.

[5] Upreti BR. Knowledge-driven approaches for financial news analytics. Network theory and agent-based modeling in economics and finance. 2019. p. 375–404.

[6] Gupta R, Gill NS. Prevention of financial statement fraud using data mining. Int J Comput Sci Inf Secur. 2012;10(4):55.

[7] Sharma S, Upadhyay R, Das S. Deep learning-based early warning system for bankruptcy risk in Indian MSMEs: A feasibility study. In: International Conference on Artificial-Business Analytics, Quantum and Machine Learning. 2023.

[8] Kotsiantis S, et al. Predicting fraudulent financial statements with machine learning techniques. In: Hellenic Conference on Artificial Intelligence. 2006.

[9] Robles-Granda PD, Belik IV. A comparison of machine learning classifiers applied to financial datasets. In: Proceedings of the World Congress on Engineering and Computer Science. 2010.

[10] Wei-Yang Lin, Ya-Han Hu, Chih-Fong Tsai. Machine Learning in Financial Crisis Prediction: A Survey. IEEE Trans Syst, Man, Cybern C. 2012;42(4):421–36. doi: 10.1109/tsmcc.2011.2170420

[11] Liu M, Gao R, Fu W. Analysis of Internet Financial Risk Control Model Based on Machine Learning Algorithms. Journal of Mathematics. 2021;2021:1–10. doi: 10.1155/2021/8541929

[12] Altman EI. FINANCIAL RATIOS, DISCRIMINANT ANALYSIS AND THE PREDICTION OF CORPORATE BANKRUPTCY. The Journal of Finance. 1968;23(4):589–609. doi: 10.1111/j.1540-6261.1968.tb00843.x

[13] Ohlson J. Financial ratios and the probabilistic prediction of bankruptcy. J Account Res. 1980.

[14] Tibshirani R. Regression shrinkage and selection via the lasso. J R Stat Soc Ser B Stat Methodol. 1996;58(1):267–88.

[15] Jolliffe I. Principal component analysis for special types of data. Springer. 2002.

[16] Schumaker R, Chen H. Textual analysis of stock market prediction using breaking financial news: the azfin text system. ACM Trans Inf Syst. 2009;27(2):1–19.

[17] Das S, Chen M. Yahoo! for Amazon: sentiment extraction from small talk on the web. Manag Sci. 2007;53(9):1375–88.

[18] Bujaki M, Mansurov A, McConomy B. The content, evolution, and determinants of COVID‐19 disclosures in Canadian financial statements and MD&A documents: an impression management perspective. Canad J Adm Sci. 2024.

[19] Liao S. Divergent investor perspectives and volatility risk--research based on stock bar public opinion data. In: SHS Web Conf. EDP Sciences. 01010.

[20] Liu W, Jiang Q, Jiang H. Stock forum comment sentiment analysis method based on finbert-cnn. Integr Technol. 2022;11(1):27–39.

[21] Li C, Jia H, Zhao G. Credit risk warning of listed companies based on information disclosure text: empirical evidence from Chinese annual reports’ management discussion and analysis. Chin J Manag Sci. 2023;31(02):18–29.

[22] Huang B, Yao X, Luo Y. Improving financial distress prediction using textual sentiment of annual reports. Ann Oper Res. 2023;330(1):457–84.

[23] Sun J, Li N, Zhao M. Media negative reporting and corporate financial distress warning: based on text analysis and machine learning. J Finance Econ. 2023;09:80–90.

[24] Ravisankar P, et al. Detection of financial statement fraud and feature selection using data mining techniques. Decis Support Syst. 2011;50(2):491–500.

[25] Hong W, Wang X, Feng H. Research on financial statement fraud detection of listed companies based on the logistic regression model. Chin J Manag Sci. 2014;22(S1):351–6.

[26] Eweoya IO, et al. Fraud prediction in bank loan administration using a decision tree. In: J Phys Conf Ser. 2019.

[27] Cao D, Liu B. Svm model for financial fraud detection. J Northeastern Univ (Nat Sci). 2019;40(2):295.

[28] Xiong T. The analysis of influence mechanism for internet financial fraud identification and user behavior based on machine learning approaches. Int J Syst Assur Eng Manag. 2022;13(3):996–1007.

[29] Dechow PM, Ge W, Schrand C. Predicting material accounting misstatements. Contemp Account Res. 2011;28(1):17–82.

[30] Ye H, Xiang L, Gan Y. Detecting financial statement fraud using random forest with SMOTE. In: IOP Conf. Ser. Mater. Sci. Eng. 2019. doi: 10.1088/1757-899X/621/5/052001

[31] Chowdhury N, Cai X, Luo C. Boostmf: boosted matrix factorization for collaborative ranking. In: Machine Learn Knowl Discov Databases: Eur Conf. 2015.

[32] Breiman L. Bagging predictors. Mach Learn. 1996;24(2):123–40. doi: 10.1007/bf00058655

[33] TANG W. Bagging-Based Selective Clusterer Ensemble. Journal of Software. 2005;16(4):496. doi: 10.1360/jos160496

[34] Zhang C, Zhang J. A review of selective ensemble learning algorithms. J Comput Sci Technol. 2011;34(8):1399–410.

[35] Shi J, Cheng L, Niu J. Financial crisis prediction of listed companies based on RS-bag classifier ensemble technology. Math Stat Manag. 2013;32(5):941–50.

[36] Bao Y, et al. Detecting accounting fraud in publicly traded US firms using a machine learning approach. J Account Res. 2020;58(1):199–235.

[37] Hu X, Tang L. Industrial internet intrusion detection method based on optimized lightweight gradient boosting machine. J Netw Inf Secur. 2023;9(02):46–55.

[38] Shimin LEI. An xgboost-based system for financial fraud detection. In: E3S Web Conf. 2020.

[39] Zhang L, Liu J, Tian D. Application of financial early warning based on stacking-bagging-vote multi-source information fusion model. Comput Appl. 2022;42(1):280–6.

[40] Sigletos G, et al. Combining information extraction systems using voting and stacked generalization. J Mach Learn Res. 2005.

[41] Park Y, Ren J. Malicious webpage detection method based on stacking. Comput Appl. 2019;39(04):1081–8.

[42] Ding L, Luo P. P2P online lending default risk early warning research based on stacking ensemble strategy. Invest Res. 2017;36(04):41–54.

[43] Zheng Y, Wang C. Credit bond default prediction model based on ensemble algorithm and its influencing factors. Financ Econ. 2023;10:18–27.

[44] Zhang X, Shi Y. Research on financial fraud detection based on meta-learning. J Manag Sci. 2023;26(10):95–113.

[45] Aghakhani S. An effective LmRMR for financial variable selection and its applications. In: 2017 IEEE International Conference on Information Reuse and Integration (IRI). 2017.

[46] Ye Z. Financial anomaly data extraction method based on random forest. J Huaiyin Norm Univ (Nat Sci Ed). 2024;23(01):13–9.

[47] Lu Z, Zhang J. Credit risk prediction of listed companies based on SMOTE Tomek-RFE-MLP algorithm. Syst Sci Math. 2022;42(10):2712–26.

[48] Wang L, Wu C. Financial crisis early warning research based on self-organizing map and fuzzy membership degree. Oper Res Manag. 2017;26(12):119–25.

[49] Wen Y, Wang J. Risk early warning system for financial holding companies based on FA-BPNN: A case study of the United States and Taiwan. Int Finance Res. 11:37–46.

[50] Zhao Q, Xu W, Ji Y. Financial distress prediction based on convolutional neural networks: Image visualization of company financial reports. J Manag Eng. 2024.

[51] Li S, He D, Lin D. Financial crisis prediction based on artificial bee colony optimized recurrent neural network. J Nanjing Univ Sci Technol. 2022;46(04):427–33.

[52] Cao C, Chen Y, Zhou L. Financial fraud detection of listed companies based on deep learning and text sentiment. Comput Eng Appl. 2024;60(04):338–46.

[53] Jones S, Johnstone D, Wilson R. An empirical evaluation of the performance of binary classifiers in the prediction of credit ratings changes. Journal of Banking & Finance. 2015;56:72–85. doi: 10.1016/j.jbankfin.2015.02.006

[54] Liu Y, Wu B, Zhang M. Design and application of financial fraud detection models for listed companies: Based on emerging machine learning algorithms. J Quant Econ Tech Econ Res. 2022;39(07):152–75.

[55] Verma V. A comprehensive guide to feature selection using wrapper methods in python. 2020.

[56] Faris H, Abukhurma R, Almanaseer W, Saadeh M, Mora AM, Castillo PA, et al. Improving financial bankruptcy prediction in a highly imbalanced class distribution using oversampling and ensemble learning: a case from the Spanish market. Prog Artif Intell. 2020;9(1):31–53.

[57] Lin X, Ren C, Li et al. Eucalyptus plantation extraction based on relief f-rfe feature selection. Surv Map Sci. 2023;48(10):107–15.

[58] Ben Jabeur S, Stef N, Carmona P. Bankruptcy prediction using the XGBoost algorithm and variable importance feature engineering. Comput Econ. 2022.

[59] Perboli G, Arabnezhad E. A machine learning-based DSS for mid and long-term company crisis prediction. Expert Syst Appl. 2021.

[60] AI-Milli N, Hudaib A, Obeid N. Population diversity control of genetic algorithm using a novel injection method for bankruptcy prediction problem. Math. 2021;9(8):823.

[61] Tsai C, Sue K, Hu Y, Chiu A. Combining feature selection, instance selection, and ensemble classification techniques for improved financial distress prediction. J Bus Res. 2021;130:200–9.

[62] Altman EI, Balzano M, Giannozzi A, Srhoj S. Revisiting SME default predictors: the omega score. J Small Bus Manag. 2022;61(6):2383–417.

[63] Du X, Li W, Ruan S, Li L. CUS-heterogeneous ensemble-based financial distress prediction for imbalanced dataset with ensemble feature selection. Applied Soft Computing. 2020;97:106758. doi: 10.1016/j.asoc.2020.106758

[64] Lin S-J, Hsu M-F. Incorporated risk metrics and hybrid AI techniques for risk management. Neural Comput & Applic. 2016;28(11):3477–89. doi: 10.1007/s00521-016-2253-4

[65] Sulistiani I, , Nugraheni M. Comparison of bankruptcy prediction models using support vector machine and artificial neural network. In: 2022 11th Electrical Power, Electronics, Communications, Controls and Informatics Seminar (EECCIS). 2022. 316–21.

[66] Wang H, Liu D, Pu G. Nuclear reconstructive feature extraction. Neural Comput & Applic. 2017;31(7):2649–59. doi: 10.1007/s00521-017-3220-4

[67] Wu X, Gao W. Fault detection of JTC trackside equipment based on tSNE multi-feature fusion. J Rail Sci Eng. 2024;21(03):1244–55.

[68] Xiang Y, Yan H, Yu X. Non-invasive load identification based on feature selection and improved self-organizing neural networks. J China Electr Eng Soc. 42(S1):106–14.

[69] Zhao H, Zou D, Xue W. Concept drift malware classification optimization based on BERT and autoencoders. J Softw.

