# Supplementary material for: Research on the financial early warning models based on ensemble learning algorithms: Introducing MD&A and stock forum comments textual indicators
Source: PLoS One. 2025 May 22;20(5):e0323737. doi: 10.1371/journal.pone.0323737 (PMC12097627; doi:10.1371/journal.pone.0323737)
Supplement: S1 Table — (DOCX) [file pone.0323737.s001.docx]

**S1 Table. Primary financial warning identification indicators (financial and nonfinancial indicators)**

| Dimension | Indicator name | Formula |
| --- | --- | --- |
| $X1$ | $Flow ratio$ | $\frac{Current Assets}{Current Liabilities}$ |
| $X2$ | $Speed ratio$ | $\frac{Current Assets-Inventory}{Current Liabilities}$ |
| $X3$ | $Cash ratio$ | $\frac{Cash-Cash Equivalents}{Current Liabilities}$ |
| $X4$ | $Asset-liability ratio$ | $\frac{Total Liabilities}{Total Assets}$ |
| $X5$ | $Equity multiplication$ | $\frac{Total Assets}{Shareholders^{'}Equity}$ |
| $X6$ | Debt-to-Equity ratio | $\frac{Total Liabilities}{Shareholders^{'}Equity}$ |
| $X7$ | $Account receivable$  $turnover rate$ | $\frac{Net Sales}{Average Accounts Receivable}$ |
| $X8$ | $Inventory turnover rate$ | $\frac{Cost of Goods Sold}{Average Inventory}$ |
| $X9$ | $Accounting rate of$  $accounts payable$ | $\frac{Cost of Goods Sold}{Average Accounts Payable}$ |
| $X10$ | $Cash turnover$ | $\frac{Net Sales}{Average Cash Balance}$ |
| $X11$ | $Current asset turnover ratio$ | $\frac{Net Sales}{Average Current Assets}$ |
| $X12$ | $Total asset turnover rate$ | $\frac{Net Sales}{Average Total Assets}$ |
| $X13$ | $Share shareholder equity$  $turnover rate$ | $\frac{Net Sales}{Average Shareholder Equity}$ |
| $X14$ | Operating capital  turnover rate | $\frac{Net Sales}{Average Operating Capital}$ |
| $X15$ | $Asset remuneration$ | $\frac{Net Profit}{Average Total Assets}$ |
| $X16$ | $Net interest rate$ | $\frac{Interest Income-Interest Expense}{Total Loans or Assets}$ |
| $X17$ | $Net interest rate on$  $mobile assets$ | $\frac{Interest Income-Interest Expense}{Total Mobile Assets}$ |
| $X18$ | $Net asset yield$ | $\frac{Net Income}{Net Assets}\times100\%$ |
| $X19$ | $Operating profit margin$ | $\frac{Operating Profit}{Operating Revenue}\times100\%$ |
| $X20$ | $Fixed asset growth rate$ | $\frac{\begin{aligned} Fixed Assets at End of Period- \\ Fixed Assets at Begining of Period \end{aligned}}{Fixed Assets at Begining of Period}\times100\%$ |
| $X21$ | $Total asset growth rate$ | $\frac{\begin{aligned} Total Assets at End of Period- \\ Total Assets at Begining of Period \end{aligned}}{Total Assets at Begining of Period}\times100\%$ |
| $X22$ | $Operating income growth rate$ | $\frac{\begin{aligned} Current Period Operating Income- \\ Previous Period Operating Income \end{aligned}}{Previous Period Operating Income}\times100\%$ |
| $X23$ | $Sustainable growth rate$ | $\frac{Return on Equity \times Retention Ratio}{1- Return on Equity \times Retention Ratio}$ |
| $X24$ | Net profit growth rate | $\frac{\Delta Net Profit}{Previous Period Net Profit}\times100\%$ |
| $X25$ | Cash Content of Operating  Revenue | $\frac{Cash Flow from Operating Activities}{Operating Revenue}\times100\%$ |
| $X26$ | All Cash Recovery Rate | $\frac{Total Cash Recovered}{Total Operating Revenue}\times100\%$ |
| $X27$ | $Operating index$ | $\frac{Current Assets}{Current Liabilities}$ |
| $X28$ | $Cash reinstation ratio$ | $\frac{\begin{aligned} Capital Expenditure+ \\ Cash Flow from Operations-Dividends Paid \end{aligned}}{Cash Flow from Operations}$ |
| $X29$ | $Earnings per share$ | $\frac{Net Income-Preferred Dividends}{Weighted Average Shares Outstanding}$ |
| $X30$ | Revenue$per share$ | $\frac{Revenue}{Weighted Average Shares Outstanding}$ |
| $X31$ | Net assets per share | $\frac{Shareholders'Equity}{Weighted Average Shares Outstanding}$ |
| $X32$ | Net profit cash and net content | $\frac{Net Operating Cash Flow}{Net Profit}\times100\%$ |
| $X33$ | Net profit cash net content | $(\frac{Net Operating Cash Flow}{Net Profit}-1)\times100\%$ |
| $X34$ | $\mathrm{Net}$ratio | $\frac{Stock Price}{Net assets Per Share}$ |
| $X35$ | P / E ratio | $\frac{Market Capitalization}{Net Profit}$ |
| $X36$ | $Types of audit opinions$ | 1 represents standard audit opinions. 0 represents non-standard audit opinions. |
| $X37$ | $Whether the top ten shareholders$  $are associated$ | 3 indicates that there is an association among the top ten shareholders. 2 indicates that there is an association between some of the shareholders. 1 indicates that there is no association among the top ten shareholders. |
| $X38$ | $The chairman and general$  $Manager concurrently appoint$  $the situation$ | 2 means concurrent positions. 1 means not concurrent |
| $X39$ | $Equity concentration$ | total shareholding percentage of the top ten shareholders |
| $X40$ | $Equity nature$ | 1 means non-state-owned enterprise;  0 means a state-owned enterprise |
| $X41$ | $The proportion of circulation$  $\mathrm{shares}$ | $\frac{Circulating Share Capital}{Total Share Capital}$ |
| $X42$ | $Tobin Q value$ | $\frac{Market Value of the Firm}{Replacement Cost of Assets}$ |
| $X43$ | The number of executives holding shares | Count of executives who own shares |
| $X44$ | Whether there are defects in internal control | 1 indicates the existence of internal control defects. 0 indicates no internal control defects. |
| $X45$ | The proportion of independent directors | $\frac{the Number ofIndependent Directors}{the Number of Directors}$ |
| $X46$ | Institutional supervision | 1 indicates institutional investors present. 0 indicates no institutional investor supervision. |
| $X47$ | Investor income | $Dividends+Capital Gains+Interest Income$ |
| $X48$ | Corporate value multiple | $\frac{Enterprise Value}{EBITDA}$ |
| $X49$ | Financial leverage | $\frac{Total Debt}{Total Assets}$ |
| $X50$ | Operating leverage | $\frac{Percentage Change in EBIT}{Percentage Change in Sales}$ |
| $X51$ | Combined leverage | $\frac{Percentage Change in EPS}{Percentage Change in Sales}$ |
